# Antimicrobial Activity of *Peganum Harmala L.* on *Streptococcus mutans *Compared to 0.2% Chlorhexidine

**Published:** 2016-09

**Authors:** Mohammad Motamedifar, Hengameh Khosropanah, Shima Dabiri

**Affiliations:** 1 Shiraz HIV/AIDS Research Center, Shiraz University of Medical Sciences, Shiraz, Iran.; 2 Dept. of Periodontics, School of Dentistry, Shiraz University of Medical Sciences, Shiraz, Iran.; 3 Student Research Committee, School of Dentistry, Shiraz University of Medical Sciences, Shiraz, Iran.

**Keywords:** *Peganum Harmala L.  *, *Streptococcus Mutans  *, Chlorhexidine, Cytotoxicity

## Abstract

**Statement of the Problem:**

Dental caries is one the most prevalent diseases that affects humans throughout their lives. *Streptococcus mutans* (*S. mutans*) is recognized as the most important microorganism during tooth cariogenicity. Reducing this germ in oral cavity can reduce the rate of tooth decays in humans.

**Purpose:**

The present study compared the antimicrobial activity of ethanolic extract of *Peganum harmala* L. seeds and 0.2% chlorhexidine on *S. mutans*.

**Materials and Method:**

Agar diffusion technique and micro broth dilution method were employed to test the antimicrobial effects of these two agents on *S. mutans*. Moreover, the cytotoxicity of ethanolic extract of *P. harmala* was studied on Vero cells by MTT (thiazolyl blue tetrazolium dye) colorimetric method. The data were analyzed with descriptive methods.

**Results:**

Concentrations of 50, 25, and 12.5 mg/mL of the extract made inhibition zones of bacterial growth around the wells; but, lower concentrations could not inhibit the growth of *S. mutans*. Besides, the antimicrobial effect of 0.2% chlorhexidine was more than 50 mg/mL of the extract. Minimum inhibitory concentration (MIC) of the extract on *S. mutans* was 1.83±0.6 mg/mL and minimum bactericidal concentration (MBC) was 4.3±1 mg/mL. The MIC and MBC for 0.2% chlorhexidine were reported to be 0.19 mg/mL, and 0.78 mg/mL, respectively. The extract concentrations more than 0.5 mg/mL were toxic and caused more than 50% Vero cell death.

**Conclusion:**

Despite the remarkable antimicrobial effects of high concentrations of *P. harmala* on *S. mutans*, high cell toxicity of this plant would restrict its *in vivo* therapeutic use.

## Introduction

Dental caries is one of the most common diseases that humans are afflicted with throughout their lives. As the incidence of dental caries is increasing in developing countries, it is very essential to apply new methods to decrease this disease.[1] *Streptococcus mutans* (*S.mutans*) is one of the most important factors that lead to caries formation.[2] *S. mutans* together with other microorganisms produce different glucans and acidificate dental plaque. As a result, the calcified structure of tooth is destroyed and dental caries would occur. Currently, chemical elimination of dental plaque by using disinfectants and mouthwashes to help the mechanical methods is worthy.[3]

 Many plants defend themselves against herbivores, microorganism, and insects by producing some secondary metabolites. The antimicrobial effects of these herbal products can be used for many remedial purposes.[4] *Peganum harmala* L. (*P. harmala*) is a small genus of zygophylacess family which widely grows in steppe areas and sandy soils in Asia, Middle East, North Africa, Australia and America.[5] *P. harmala* is called 'Espand' in Iran and the seeds are burned to disinfect the environment.[4]

 Different pharmacological and therapeutic effects of *P. harmala *are investigated in many studies.[4, 6-8]The important remedial aspects of this herb are its as antitumoral, antibacterial, antifungal, anti-parasitic, anti-nociceptive, anti-inflammation, vaso-relaxant, and anti-spasmodic activities. This plant is also used for diabetes, jaundice, asthma, dermatitis and many other illnesses.[4, 6-8] Pharmacological properties of *P. harmala* are attributed to the production of alkaloids in different parts of the herb. The most important alkaloids in *P. harmala* are beta-carbonyl derivations such as harmalin, harmalol, peganine, isopeganine, deoxyisopeganine; as well as quinazoline derivations such as vasicinone, vasicine, and deoxyvasicinone.[5, 9] Most alkaloids of this herb are derived from the seeds and roots. Harmalin is the best-known alkaloid in several researches that was studied.[5, 10] The ability to intercalate DNA and the resulting frame shift mutations are among the etiological factors for antibacterial effects of this plant.[11]

Ethanolic extract of *P. harmala* can restrain the growth of *Streptococcus pyogenes*, *Staphylococcus aureus* and *Staphylococcus epidermidis*.[4] Additionally, the alcoholic and aqueous extract of *P. harmala* can reduce the growth of *lactobacillus* and Candida albicans, two common microorganisms in oral cavity.[12] Moreover, *Bacillus subtilis* and *Proteus vulgaris* are the most sensitive bacteria to the metabolites derived from this plant.[13]

 Chlorhexidine is the most effective mouthwash, used to reduce dental plaque germs. It has bacteriostatic and bactericidal effects with suitable durability in oral cavity. It is more effective on *S. mutans* than *lactobacillus*; however, several side effects on tooth and oral mucosa limit the long-term usage of this mouthwash.[14] Tooth discoloration, changing in taste, increasing supra-gingival calculus, allergy, and oral lesions are most common side effects of chlorhexidine. Tooth discoloration is the most common consumer complaint.[14-15]

It seems essential to investigate other antimicrobial agents that have inhibitory effect on relevant microorganisms in oral and dental diseases with fewer side effects compared to chlorhexidine. This study aimed to investigate the antimicrobial effect of ethanol extract of *P. harmala* on *S. mutans*. In addition, the cytotoxicity of this extract was evaluated on Vero cells. 

## Materials and Method

This experimental study was done in Department of Bacteriology and Virology of Shiraz University of Medical Sciences, Shiraz, Iran. Standard strain of *S. mutans* (ATCC 35668; PTTCC 1683) was obtained from Iranian Research Organization for Science and Technology, Tehran, Iran.

Different parts of *P. harmala* were collected from areas around Shiraz, Fars province, Iran-2014. Herbarium of the plant was confirmed and a voucher number (11854) was received from Fars Integrated Agricultural Complex. All parts of the plant were dried at room temperature for 1 week. Then, the seeds were separated from other parts of the plant and were ground into fine powder. One gram of the powder was soaked in 500 mg of 96% ethanol for 48 hours at room temperature. The obtained extract was filtered by filter paper (Wattman No.1) and left in room temperature for 48 hours for drying and evaporating of the solvent. Finally, it was kept in refrigerator until used.[1]

 Agar diffusion technique was used to determine the antimicrobial effect of ethanolic extract of *P. harmala* on *S. mutans*. First, *S. mutans* was cultured in blood agar for 24 hours. Then, a suspension with 0.5 McFarland turbidity (1.5x10[8] cfu/mL) was adjusted in BHI (brain heart infusion broth). By using a sterile cotton swab, the bacterial suspension was applied on MHA (Muller Hinton agar) with 5% blood sheep. The holes were prepared by using cork borer in MHA (6mm in diameter and 4mm in height with 25mm distance). Each well was filled by 100µL of different concentrations of ethanolic extract of *P. harmala* (3.125-50mg/mL). The central hole was filled with 100µL of 0.2% chlorhexidine as positive control. The plates were incubated at 37°C in 5% incubator for 24 hours. Finally, inhibition zones were measured in millimeter.[2]

To determine the minimum inhibitory concentration (MIC) and minimum bactericidal concentration (MBC) of ethanolic extract of *P. harmala*, micro broth dilution method was used. First, 100µL of BHI were added to each well of 96-well microtiter plates. Then, 100µL of highest concentration of extract (50 mg/mL) was added to the first well. Other wells were filled with lower concentrations until the tenth well respectively (0.048- 50mg/mL). One well contained just BHI medium as negative control and one well contained BHI and bacterial suspension as the control with bacterium. The plates were incubated at 37°C in 5% CO2 incubator for 24 hours. The first well in those series that showed no sign of visible growth of bacteria was considered as MIC. The MBC was determined by culturing 10µL of contents of wells that did not show any sign of bacterial growth on blood agar. The plates were incubated for 24 hours at 37°C in 5% CO2 incubator. The least concentration that inhibited colony forming of *S. mutans* on agar was considered as MBC. All data were expressed as descriptive analysis (mean and standard deviation). 

To determine the toxicity of ethanolic extract of *P. harmala*, Vero cell line (kidney fibroblast of green African monkey) was used. The Vero cells were cultured in DMEM (Dulbecco's modified eagle medium) containing 14% bicarbonate sodium, 7% fetal bovine serum, 100 unit/mL penicillin, 100µg/mL streptomycin sulfate, 0.25 µg/mL amphotericin B. They were incubated at 37°C in 5% CO2 incubator for 48 hours. The mono layer cell culture was washed with PBS (phosphate buffered saline), and then 1mL of Trypsin-Versene solution was added to separate the Vero cells from each other. The cell count was adjusted to 2×10[5] cells in 1mL of DMEM. Then 0.1mL of diluted cell suspension was added to each well of 96-well micro titer plates. After 3 days, monolayer of Vero cells was formed and washed with PBS. Then, 1 mL of serial concentration of ethanolic extract of *P. harmala *in DMEM with 2% fetal bovine serum was added to each well. Six wells containing medium without extract were considered as control. The plate was incubated at 37°C in 5% CO2 incubator for 72 hours. Having washed the wells with PBS, 50µL of MTT (thiazolyl blue tetrazolium dye) was added to each of them and they were incubated for 2 hours. Next, 50 µL of dimethyl sulfoxide (DMSO) was set up in each well and incubated again for 30 minutes. The absorbance was measured, using a microplate reader at 405-450 nm. Absorbance values that were lower than the controls indicated reduction in cell growth. The concentration required to reduce the cell growth to less than 50% (CC50) was determined by using regression chart. 

## Results

**Figure 1:**
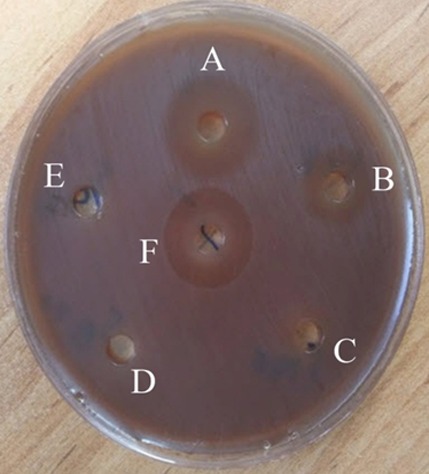
Sensitivity of *S. mutans* to different concentrations of *P. harmala* in well diffusion technique, (A) 50 mg/mL (B) 25 mg/mL (C) 12.5 mg/mL (D) 6.25 mg/mL (E) 3.125 mg/mL (F) 0.2% chlorhexidine

The anti-bacterial effects of 3.125-50 mg/mL concentrations of ethanolic extract of *P. harmala* and 0.2% chlorhexidine were checked out by using well diffusion technique (Figure 1). 

The results showed that 50 mg/mL concentration of extract, which created zones with the mean diameter of 17.8 mm, had the most antibacterial effect on *S. mutans* compared to other concentrations. The mean inhibition zones for 25 mg/mL were 11 mm and for 12.5 mg/mL was 8 mm. But 3.125 and 6.26mg/mL concentrations could not create inhibition zones. The mean diameter of zones created by 0.2% chlorhexidine was 21.2mm (Table 1). 

**Table 1:** The effect of ethanolic extract of *P. harmala* on *S. mutans* by well diffusion technique

**Extract Concentration ** **(mg/mL)**	**Inhibition zone** **mean± standard deviation (mm)**
50	17±2
25	11±3
12.5	4±5
6.25	0±0
3.125	0±0
Chlorhexidine 0.2%	21±1

**Figure 2:**
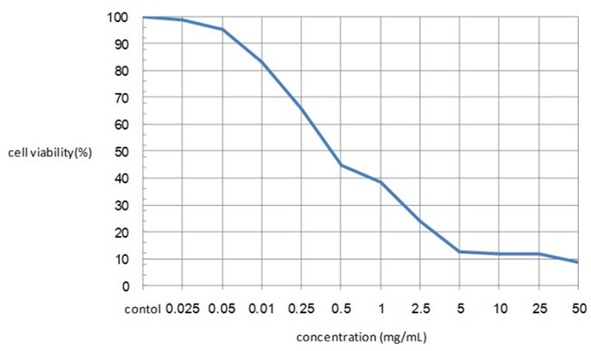
Viability of Vero cells in different concentrations of ethanolic extract of *P. harmala*

MIC of extract on* S. mutans* was 1.83±0.6 mg/mL and MBC was 4.3±1 mg/mL. MIC and MBC for 0.2% chlorhexidine were detected as 0.19 mg/mL and 0.78 mg/mL, respectively. Concerning the results of MTT method, all concentrations of more than 0.5 mg/mL of the extract caused cell death over 50% (Figure 2).

## Discussion

This study investigated the effect of *P. harmala* seeds on the growth of *S. mutans *as the most important microorganism involved in dental caries. Numerous medicinal and therapeutic properties of this plant were studied in many investigations. The presence of abundant sources of alkaloid in different parts of *P. harmala*, especially in roots and seeds, is recognized as the cause of these effects.[1] These compounds are produced to protect the plant against microorganisms, herbivores, and insects.[2] Since the main components of *P. harmala* are aromatic and fat soluble, presence of ethanol or methanol as the solvent during extraction can derive more metabolites extracted from the plant. So, in this study, 98% ethanol was used during the extraction and evaporated after the procedure was done.[3]

Concentrations of 12.5 and 50 mg/mL have caused growth inhibition of *S. mutans*. However, concentration of 50 mg/mL had the highest inhibitory effect compared to other concentrations. It can be concluded that by increasing the concentration, with increased level of alkaloids, the power of the extract to inhibit the growth of *S. mutans* increases.[4] Although the inhibition zones created by 0.2% chlorhexidine were more than those caused by 50 mg/mL extract, it is likely that higher concentrations of the extract can have similar or greater inhibitory effect than 0.2% chlorhexidine on *S. mutans*. The presence of agar as intermediate substance in agar well diffusion technique can restrict the ability of extract to spread and make appropriate prevention of microorganisms’ growth. In evaluating the function of extract with more accurate method as micro broth dilution assay, it was observed that the concentrations higher than 5.4 mg/mL of the extract had the ability to inhibit the growth of *S. mutans.*

In a study, the alcoholic and aqueous extract of *P. harmala* seeds had a limiting effect on *lactobacillus*, as one of the important microorganisms in making dental caries. In this study, ethanol extract of the herb had a greater inhibitory effect than the aqueous extract.[4]

Darabpour *et al*.[4] reported that the alcoholic extract of *P. harmala* in 50-400 mg/mL concentrations had inhibitory effect on gram positive germs like *Bacillus cereus*, *S. epidermidis*, *Streptococcus pyogenes*, *S. aureus* and gram negative germs like *Salmonella typhi*, *Escherichia coli*, *Pseudomonas aeroginosa* and *Klebsiella pneumoniae*. So, using this agent in oral cavity can also affect gram negative bacteria.

Another study investigated the sensitivity of *K. pneumoniae* to alcoholic extract of *P. harmala* in 25-100 mg/mL compared with choice antibiotics in treatment of *K. pneumoniae*. The MIC and MBC results were in line with our results in this study. In addition, the alcoholic extract of *P. harmala* has synergistic effect with gentamicin and imipenem against *K. pneumonia*.[5] According to Arshad *et al*.’s research,[3] the ethanolic extract of *P. harmala* prevented the growth of nineteen bacterial species isolated from poultry.

Despite the numerous therapeutic properties, this plant has a great cytotoxicity. Systemic use of *P. harmala* in high concentrations on animal cases caused many severe side effects such as cardiovascular, nervous, hepatic, and gastro intestinal complications. Toxicity of this herb is attributed to its inhibitory effect on monoamine oxidase (MAO), and the ability to intercalate into DNA and cause frame shift mutation.[16] According to the result of MTT colorimetric method in the present study, up to 50% of cultured Vero cells could survive only in concentrations less than 0.5 mg/mL of this extract; while in all previous studies, the emphasize was only on antibacterial properties of *P. harmala*, and the high concentrations of the herb extracts which were used.[4, 10, 12] Although high concentrations of *P. harmala* extract have more inhibitory effect on different microorganisms compared to positive controls, none of these concentrations can be used as an antibacterial agent *in vivo*. It is also reported that ethanolic extract of *P. harmala* had cytotoxicity effects on human embryonic skin fibroblast, epithelial carcinoma of uterus cervix, and oral epithelial carcinoma by MTT method.[17]

Corresponding studies are suggested to investigate the cytotoxicity of this herb on oral epithelial cells for more accurate results. Moreover, controlled *in vivo* studies seem essential to be conducted on the use of this extract per se or in combined preparations as mouth wash.

## Conclusion

In conclusion, ethanolic extract of *P. harmala* can inhibit the growth of *S. mutans*. Despite the higher inhibitory effect of 0.2% chlorhexidine compared to 50 mg/mL of this extract, other studies indicate that higher concentrations of *P. harmala* extract can have similar or greater inhibitory effect against microorganisms. However, high cell toxicity of this extract on cells limits the use of this plant as an antimicrobial agent (like mouthwash) in oral cavity. Even if limited to low concentrations, either lonely or in combined preparations, its application might be more useful for resistant bacteria compared to the routine available mouthwashes. Further antimicrobial and cytotoxicity studies on animals or human subjects are needed to obtain more accurate and applicable results. 
